# The Frequency of Tuberculosis in Cows Slaughtered for Food

**Published:** 1882-10

**Authors:** 


THE FREQUENCY OF TUBERCULOSIS IN COWS SLAUGHTERED FOR FOOD is a question of much importance to have definitely settled. In another paragraph we have referred to some facts bearing upon this point. We stated that among 12,269 cattle slaughtered in Augsburg in 1881, 246, or 2.01 per cent., were found tuberculous. Among

24,901 calves under the age of four weeks, there was found no tuberculosis at all.

The age of the tuberculous cattle was, with the exception of twenty-three, over three years.

The seat of the tubercles was in 68 cases in the lungs and pleura, in 142 cases in the lungs alone, and in 37 cases in the pleura alone; in 59 cases there were also tubercles in the liver.

Of these infected cattle the flesh of only 18 was condemned as unfit for use. During the year six of the cows thus found tuberculous had for a long time been used as milch cows for the city.

